# Hyperlipidemia as an Instigator of Inflammation: Inaugurating New Approaches to Vascular Prevention

**DOI:** 10.1161/JAHA.112.000497

**Published:** 2012-02-20

**Authors:** Paul M. Ridker

**Affiliations:** Center for Cardiovascular Disease Prevention, Brigham and Women's Hospital, Harvard Medical SchoolBoston, MA

For much of the past quarter century, 2 broadly competing scientific views have dominated translational research concerning atherogenesis and atherothrombosis. On one side has been a predominantly lipid-centric view in which low-density lipoprotein (LDL) cholesterol, a proven causal factor in atherosclerosis, has been viewed as the major if not sole determinant of disease initiation and progression. Pioneering descriptions of the role played by the LDL receptor in human disease and the remarkable success of statin therapy provide emblematic support for this scientific viewpoint and are milestones in the history of cardiovascular medicine.^1^ Ongoing research into agents that reduce proprotein convertase subtilisin/kexin type 9 (PCSK9) activity^2^ or that inhibit the intestinal Niemann-Pick C1-like protein 1 (NPC1L1) cholesterol transporter^3^ represent current expressions of the established view that ever lower levels of LDL cholesterol are likely to be beneficial, and that pharmacologic inhibition of cholesterol on top of statin therapy might again transform medical practice. However, there are paradoxes in the LDL literature that have long puzzled investigators including observations that LDL cholesterol is only a modest predictor of vascular risk in the general population; that most myocardial infarction and stroke events occur among those with relatively low LDL cholesterol levels; that not all agents that reduce LDL cholesterol reduce vascular events; and that the relative risk reductions associated with statin therapy occur within weeks of drug initiation and are fully independent of the underlying level of LDL cholesterol.

These paradoxes are commonly cited to support an alternative view of atherothrombosis based fundamentally on the vascular biology of inflammation. Rather than viewing atherosclerosis solely as a lipid deposition disorder within a passive arterial wall, the inflammatory model of atherogenesis and atherothrombosis proposes that critical components of the innate and adaptive immune systems contribute heavily to atherogenesis and thus that alterations of immunity may have therapeutic potential.^4^ With regard to the “fast and blunt” innate immune response, multiple cells involved in atherosclerosis express pattern recognition receptors that can alter inflammatory signaling, and recent work suggests crucial roles for monocyte/macrophage lines as well as mast cells in human atherogenesis. With regard to the “slow but specific” adaptive immune response based on antigen presentation and biologic memory, abundant evidence has accrued implicating several specific types of antigen recognizing T cells, antibody secreting B lymphocytes, and antigen presenting dendritic cells in all stages of the atherothrombotic process.

At times, the above world views have appeared to be in competition. However, for most investigators within the translational research community, hypotheses characterized as strictly “lipid driven” or strictly “inflammation driven” present a false dichotomy. Few if any supporters of the inflammation hypothesis do not fully endorse the fundamental role of LDL cholesterol in atherogenesis, just as few if any supporters of the LDL-centric hypothesis do not recognize the pro- and anti-inflammatory effects that different lipid fractions have on vascular function. In this regard, modified lipoproteins are known to interact with scavenger receptors of the innate immune system, and direct binding of oxidized LDL to CD36 and of apolipoprotein CIII to toll-like receptor 2 is well described.^5^

In my own research group, we have long looked at the “lipid” and “inflammation” hypotheses as closely interrelated because clinical data supporting inflammation largely parallel those supporting LDL cholesterol. As examples, the magnitude of risk associated with each standard deviation increase in the inflammatory biomarker C-reactive protein (CRP) is remarkably similar to that of LDL cholesterol, and both contribute independently toward improved vascular risk prediction.^6^ Further, statins significantly reduce both LDL cholesterol and CRP, and reductions in each parameter have consistently proven to be important as determinants of overall statin efficacy.^7,8^ In the recently completed JUPITER trial, statin therapy was highly effective at lowering vascular event rates in primary prevention even among men and women with levels of LDL cholesterol well below current treatment thresholds, and this benefit occurred concomitantly with large reductions in both LDL cholesterol and CRP.^9^ An appealing view of statin therapy is that these remarkably effective agents are “2 fers” that both reduce LDL and have clinically relevant antiinflammatory properties. It is thus difficult, if not impossible, to invoke the statin literature to promote a hypothesis of atherothrombosis based solely on LDL lowering or on inflammation inhibition. Evidence that statins significantly reduce deep vein thrombosis and pulmonary embolism has made this point clear for the clinical community; there are no atherosclerotic lesions within the venous system and LDL cholesterol is at best a marginal player in the development of venous thrombosis, yet in the JUPITER trial, random allocation to rosuvastatin as compared with placebo reduced venous thromboembolism at least as much as it reduced myocardial infarction and stroke.^10^

In 2010, 2 important papers were published that provided a further glimpse of how the “lipid” and “inflammation” hypotheses may be more directly linked than previously appreciated. Each of the 2010 papers reported that the intracellular NLRP3 inflammasome critical for caspase activation and the subsequent production and secretion of mature interleukin-1β respond not only to crystalline uric acid and crystalline pyrophosphate, but also to crystalline cholesterol.^11,12^ These observations identify the very early deposition of minimally modified LDL cholesterol as an “endogenous danger signal” capable of triggering interleukin-1β and thus describe a new pathway by which cholesterol can directly induce a proinflammatory response. In addition to providing linkage between LDL cholesterol and early inflammation, the NLRP3 data are clinically relevant as interleukin-1β itself is a driver of the acute phase response. Thus, cholesterol-driven induction of the NLRP3 inflammasome provides a unifying causal pathway that helps to explain, in part, why systemic biomarkers of inflammation including CRP and interleukin-6 are elevated so many years in advance of acute coronary obstruction.

In this inaugural issue of the *Journal of the American Heart Association*, Ammirati and colleagues in Milan add importantly to this body of work by presenting data indicating that our understanding of the complex intersection between lipid biology and inflammation may also require careful cellular subphenotyping, at least as we look toward novel T-cell targets for intervention.^13^ Previous work from several laboratories has suggested a role for CD4^+^ T cells in atherosclerotic lesion formation, but it has been uncertain as to whether specific CD4^+^ T-cell subsets might have greater or lesser relevance to disease progression.

Taking advantage of polychromatic flow cytometry (which allows the simultaneous identification of multiple T-cell subphenotypes based on the expression of specific markers including CD3/CD4/CD45RO/CD45RA/CCR7/CCR5/ CXCR3/HLA-DR), Ammirati and colleagues show that several specific CD4^+^ subphenotypes of circulating effector memory T cells (T_EM_) are preferentially associated with human atherosclerosis. Specifically, in one human cohort of stable patients, the investigators present data that T_EM_ designated as CD3^+^CD4^+^CD45RA^−^CD45RO^+^CCR7^−^ were more likely to associate with common carotid intimal medial thickness and were more likely to correlate with LDL cholesterol levels than were other T-cell subphenotypes. In a second cohort, the investigators observed that T_EM_ identified as HLA-DR^+^ were more prevalent among those with chronic stable angina or acute infarction than among controls free of ischemia. Finally, in a separate set of mouse studies, the investigators report that T_EM_ identified as CD4^+^CD44^+^CD62L^−^ are increased in LDL-receptor and apolipoprotein-E deficient mice and correlate to a greater extent with aortic root lesions.

The data from Ammirati and colleagues raise intriguing issues about the complex roles played by circulating T-cell subsets in human atherosclerosis, and provide evidence that cellular subphenotyping to identify specific CD4^+^ cells that have lost CCR7 may be relevant as new targets for antiinflammatory therapy are developed. It is also worth considering how these new observations fit into a unified hypothesis of cholesterol as a potential instigating factor for inflammation and early atherogenesis. In hypercholesterolemic animal models, the CCR7 knockout is known to attenuate plaque development.^14^ Thus, as memory T cells are broadly antigen-experienced and as T_EM_-cell subsets have lost CCR7 receptors, one attractive interpretation of the current data is that cholesterol itself may be a key antigen stimulating T_EM_-cell expansion. If so, then the current data further suggest that a false distinction is being made in the clinical and investigative communities between hyperlipidemia and inflammation as separate competing processes.

A fundamental challenge for any investigative field where entrenched hypotheses dominate care is to ensure that novel avenues of investigation remain open and productive. Today within the cardiovascular community, we are blessed to have major clinical trials underway that are specifically addressing whether aggressive LDL cholesterol lowering through pathways other than 3-hydroxy-3-methylglutaryl-coenzyme A reduction can reduce vascular risk; that are specifically addressing whether high-density lipoprotein raising through cholesteryl ester transfer protein inhibition with or without concomitant LDL reduction might provide clinical benefit; and that address whether targeted inhibition of the secretory phospholipases Lp-PLA2 and sp-LA2 might improve patient outcomes. Recently, my colleagues and I have been given the opportunity to launch 2 “Cardiovascular Inflammation Reduction Trials,” one funded by industry addressing whether canakinumab (a monoclonal antibody targeting interleukin-1β) can reduce secondary event rates, and one funded by the National Heart, Lung and Blood Institute addressing whether low-dose methotrexate (a staple for the treatment of rheumatoid arthritis) might also confer cardiovascular protection. Strategies being leveraged by other investigative groups include evaluation of alternative cytokine and leukotriene inhibitors; methods to directly prevent monocyte chemotaxis; methods to inhibit mast cell function; methods to target cell proliferation, adhesion, and migration; and novel vaccine approaches with the potential to impact on antigen response and humoral immunity. All of these novel concepts deserve the support of the clinical cardiovascular community to ensure adequate patient enrollment to address fundamental hypotheses. At a minimum, trials of these agents will enhance our understanding of the intersection between lipid biology and inflammation. If we are lucky, and if the core biology holds up, one or more of these strategies may ultimately provide substantive clinical benefit for our patients.

Fifteen years ago, Professor Attilio Maseri—a pioneer in inflammation biology—wrote a commentary on an early CRP paper^15^ in which he suggested that observing a relationship between inflammatory biomarkers in currently healthy men and the risk of future myocardial infarction provided a glimpse of the “hidden side of the moon.”^16^ It is thus entirely fitting in this inaugural issue of the *Journal of the American Heart Association* that we celebrate the ongoing insights and creativity from Dr Maseri and his many productive Italian colleagues through publication of original data that continue to challenge conventional wisdom regarding the linkage between cellular inflammation, LDL cholesterol, and clinically evident atherosclerotic disease. That so many clinical cardiologists worldwide are now aggressively engaged in trials of novel lipid altering and inflammation inhibiting agents is a testament to the distance bridged by the vascular biology and lipid communities over these intervening years.
